# Utilization of the Integrated Management of Newborn and Childhood Illness (IMNCI) protocol and associated factors among health care workers in health centers of South Gondar Zone, Northwest Ethiopia: an institution-based mixed study

**DOI:** 10.3389/frhs.2024.1364661

**Published:** 2024-04-22

**Authors:** Abraham Addis Belete, Yeshambel Agumas, Asmamaw Ketemaw Tsehay, Habtamu Molla Ayele

**Affiliations:** ^1^ School of Public Health, College of Medicine and Health Science, Bahir Dar University, Bahir Dar, Ethiopia; ^2^ Maternal and Child Health Directorate, Federal Ministry of Health, Addis Ababa, Ethiopia

**Keywords:** Integrated Management of Newborn and Child Illnesses, utilization, health care workers, South Gondar Zone, Ethiopia

## Abstract

**Introduction:**

Globally, 11 million children have died before reaching their fifth birthday. The introduction of the Integrated Management of Newborn and Childhood Illness (IMNCI) protocol reduces the morbidity and mortality rates among children under the age of 5. However, the share of neonatal mortality is increasing. As a result, the United Nations has established sustainable development goals to reduce national neonatal death rates through the utilization of the Integrated Management of Newborn and Childhood Illness protocol as the main tool for 12 per 1,000 live births by 2030. However, the death rate from preventable causes has increased owing to the low utilization of the protocol.

**Objective:**

The objective of this research is to assess the utilization of the IMNCI protocol and associated factors among healthcare workers (HCW) in health centers at the South Gondar Zone, Northwest Ethiopia, in 2022.

**Methods:**

The institution-based mixed study design was conducted from November to December 2022 at the South Gondar Zone, Amhara. A total of 422 HCW were selected using a computer-generated random-number generator. Data were cleaned and entered into EpiData 3.1 software and analyzed using SPSS version 25.0. Binary logistic regression was used to identify candidates for multivariable logistic analysis with *p*-value < 0.2, and for multivariable analysis, *p*-value < 0.05, with a 95% confidence interval, was considered significant. Thematic analysis was used for the qualitative data.

**Results:**

In total, 417 respondents were included in the final analysis. The overall response rate was 98.8%, and the mean age was 30.01 years. The results showed that the proportion of IMNCI utilization was 63.1%. The odds of the utilization of IMNCI protocol among HCW who took training were 3.13 times higher than those among HCW who did not take training [adjusted odds ratio (AOR) = 3.13, 95% CI: 1.594, 6.147]. The lack of drugs reduces the utilization of the IMNCI protocol by 75.7% compared with the use of drugs (AOR = 0.243, CI: 95%:0.128, 0.464). HCW who always referred to the chart booklet during case management were 3.34 times more likely to utilize the IMNCI protocol (AOR = 3.34, 95% CI: 1.99, 5.60) compared with those who did not refer to the chart booklet.

**Conclusions and recommendations:**

The utilization of the IMNCI protocol was low. A shortage of medical consumables and equipment attitude and training were identified as factors that affected the utilization of the protocol. Therefore, the availability of necessary logistics and training for employees with regular supportive supervision and monitoring should be conducted with the integration of sectors at the district and zone levels.

## Introduction

The Integrated Management of Newborn and Childhood Illness (IMNCI) protocol is a holistic approach that focuses on the well-being of children under the age of 5. The IMNCI protocol helps to reduce death, illness, and disability and to improve the growth and development of under-five children. It is a set of preventive and curative child health services provided at the health center (HC) and hospital levels (1). In 1992, the World Health Organization (WHO), United Nations International Children's Emergency Fund (UNICEF), and other collaborators created the IMNCI protocol. In 1996, Ethiopia initiated IMNCI training and implemented the guidelines for treating under-five children in Tigray, the Southern Nation, Nationalities and Peoples Region, and Addis Ababa. In 2004, all regions of Ethiopia included the IMNCI protocol as part of their health program. The IMNCI protocol helps healthcare workers (HCW) diagnose pediatric diseases, identify exact treatments, strengthen the counseling of caretakers, and facilitate the referral system in healthcare facilities (2).

The IMNCI strategy has three components: health professional components that help HCW manage cases using the adapted guidelines. The second component is the health service component, which concerns the overall situation of health institutions for the effective management of under-five children. The other is the community component, which is related to encouraging the healthcare practices of the community and families.

The IMNCI protocol aims to promote quality case management practices based on age categories of up to 2 months and 2 months to 5 years. In the IMNCI protocol, a child's signs and symptoms help classify their disease condition in a color-coded manner, in which pink suggests hospital referral or admission, yellow indicates initiation of treatment, and green calls for home treatment. It helps clinicians identify which children require hospital care vs. home treatment. Through the IMNCI protocol, a HCW at a remote clinic or HC may determine whether a child with dehydration from diarrhea needs to be sent to a regional hospital. The utilization of this standard protocol also promotes rational drug use through the use of a limited number of essential drugs. Utilizing the IMNCI protocol improves the awareness of the caretaker on how to administer oral drugs and treat the child at home when hospital admission is not needed (3). The IMNCI protocol is considered a means to achieve sustainable development goals (SDGs), which aim to end preventable neonatal and child mortality by securing nutritional needs and access to quality care and boosting childhood development by 2030 (4). Despite this, the utilization of the IMNCI protocol faces challenges that inhibit the achievement of SDG targets regarding neonatal and child mortality, which is a concern in many countries, including Ethiopia.

Currently, the IMNCI method is crucial for reducing child mortality and morbidity. Despite the high child mortality rate, in Ethiopia especially in Amhara, studies conducted on the factors influencing the utilization of the IMNCI protocol are limited. The objective of this research is to assess the utilization of IMNCI protocol and associated factors among HCW in HCs at the South Gondar Zone, Northwest Ethiopia. Therefore, this study will be helpful to planners in planning comprehensive services for under-five children. The study findings will be useful for the community in the reduction of morbidity and mortality rates among under-five children through proper utilization of IMNCI guidelines by healthcare providers, with a focus on measuring inhibiting factors.

## Methods and materials

### Study area

The study was conducted at the South Gondar Zone, 121 km from Bahir Dar. There are 13 districts in this zone. The overall population is estimated to be 2,952,326 in 2022. A total of 399,745 children under the age of 5 are estimated to live in the zone. The zone has 10 hospitals and 96 HCs.

### Study design and study period

The institution-based mixed study was conducted among HCW in the HCs at the South Gondar Zone, Northwest Ethiopia, from November 2022 to December 2022.

### Source population and study population

#### Source population

The source population consisted of all health professionals working on the IMNCI protocol in the HCs at the South Gondar Zone, Amhara, Ethiopia.

### Study population

This study involved all health professionals working in selected districts with under-five outpatient departments (OPDs) who were present on the days of data collection.

### Eligibility criteria

#### Inclusion criteria

We included all health professionals who were working in the selected district at HCs in under-five OPDs and were present on the days of data collection.

#### Exclusion criteria

We excluded all health professionals who were not available on the days of data collection due to illness, maternity, and annual leave.

## Sample size determination

The sample size for the first objective was determined using a single population proportion formula with the following assumptions: the proportion utilization of IMNCI is 58.7% in the West Arsi Zone, Ethiopia:

n=(Zα/2)2p(1−p)/d2

n=(1.96)2(0.587)(1−0.587)/(0.05)2=372

where *n* is the required sample size; *p* is the utilization value of the IMNCI protocol in the West Arsi Zone, which is 58.7%; *z* is the value of the standard normal curve score corresponding to the given confidence interval of 1.96; and *d* is the margin of error. Since the entire population is <10,000, the required sample size will be as follows:

nf=n/(1+(n/N))

nf=372/(1+(372/824))=256

where nf is the desired sample size (population < 10,000), *n* is the desired sample size (population > 10,000), and *N* is the estimate of the population size.

After using a 1.5 design effect and adding a 10% non-response rate, the total sample size was 422 HCW.

For the second objective of the study, the most significant factor for determining the sample size from a previous study conducted in the North Shew Zone was training and another was the utilization of a chart booklet by HCW, as evidenced by research conducted in the West Arsi Zone, Ethiopia, as shown in Table 1.

**Table 1:** Sample size for the factors associated with the utilization of the Integrated Management of Newborn and Childhood Illness (IMNCI) protocol among healthcare workers (HCW) at health centers (HCs) in the South Gondar Zone, Northwest Ethiopia.

Factors	Factor group	Assumption	Sample size
Attending training	Yes	Percent of controls exposed 17.9%, Percent of cases with exposure = 39.8% Case to control ratio = 1:2, power = 80%, CI = 95%, OR = 2.6	164
HCW referring to the chart booklet	Yes	Percent of controls exposed = 16.4%, Percent of cases with exposure = 33.3% Case to control ratio = 1:2, Power = 80%, CI = 95 %, OR = 2.76	233

OR, odds ratio.

The sample size for the first objective was larger than that for the second objective (422). Therefore, the sample size for the first objective was used.

For the qualitative study, IMNCI focal persons were asked. The sample size was continued until adequate information was obtained from the participants.

## Sampling technique and procedure

**Figure 1:**
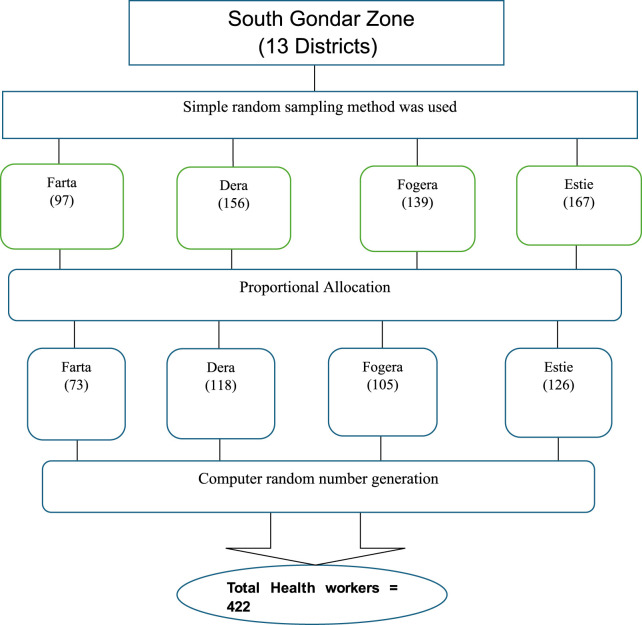
Schematic presentation of sampling procedure using the South Gondar Zone Health Department 2022 annual report.

The South Gondar Zone has 13 districts. It has 10 hospitals and 96 HCs that provide healthcare services for the local communities. The IMNCI protocol is used in all districts of the zone. Using a multistage sampling of 13 districts, four districts were selected using the lottery method. The study was mainly done on HCW working in HCs of the districts. For the quantitative study, proportional allocations were done to select the number of participants in each selected district. Simple random sampling was used to access the study participants based on the human resource registration in each district, as shown in Figure 1.

For the qualitative study, IMNCI focal persons were asked from the randomly selected districts. The participants were selected purposively. The sample size was continued until adequate information was obtained from the participants.

## Variables

Dependent variable
  Utilization of the IMNCI protocol
Independent variable
  Sociodemographic factors
    • Age
    • Gender
    • Marital status
    • Educational level
    • Religion
    • Ethnicity
  Healthcare system factors
    • Drug
    • Supervision
    • Chart booklet
    • Equipment
  Healthcare works related
    • Number of staffs
    • Training
    • Year of service
    • Staff turnover
    • Educational qualification
    • Attitude

## Data collection tools and procedure

A structured questionnaire was developed for data collection after reviewing the relevant literature. The questionnaire was prepared in English. The questionnaire was adapted from studies with similar purposes. Four nurses participated in the collection of the completed questionnaires from each woreda. They explained the purpose of the study and how to correct the completion of the semi-structured questionnaire. For the qualitative study, key informant interviews were conducted to collect the data.

## Data quality control

To ensure the quality of the data, we used properly designed data collection instruments and discussions and explanations with nurses who distributed and collected the completed questionnaires. The questionnaire was tested at the Addis Zemen Health Center, which was not included in the actual study, to ensure the quality and reliability of the questionnaire. Cronbach's alpha was 0.73. The investigator regularly checked for completeness and quality of the data.

## Data processing and analysis

The data were coded and entered into EpiData 3.1 and then exported to the SPSS version 25 statistical package. Data cleaning was performed to check for outliers and missing data. A descriptive analysis was performed for proportions and percentages to describe the study population regarding relevant variables. Descriptive data are presented in the tables and graphs. Bivariate analysis was used to identify the associated factors. Multivariable analysis was used for factors with a *p*-value < 0.2. *p*-values and 95% confidence intervals (CI) for OR were used to judge the significance of the associations. A *p*-value < 0.05 was considered a significant association. The Hosmer–Lemeshow model fitness test was used to indicate the goodness of fit of the final model, and model fitness was assured at a *p*-value = 0.425. Multicollinearity was checked using VIF, which was <10.

To analyze the data from key informant interviews, a thematic analysis was undertaken.

## Result

### Sociodemographic characteristics

In total, 417 respondents were included in the final analysis. The overall response rate was 98.8%, and the mean age of the respondents was 31.01 years, with an SD of 4.3 years. Of the study participants, 246 (59%) were male. The majority of health professionals, 227 (54.4%), were <30 years old. Approximately 185 (44.4) had <5 years of work experience, and 177 (42.4%) had work experience ranging from 5 to 10 years. Of the total, more than half of the respondents, 119 (59.2%), had served as healthcare providers for <5 years. Of the total respondents, 245 (58.8%) were diploma holders, and 165 (39.6%) were Bachelor of Science (BSc) degree holders, as shown in Table 2.

**Table 2:** Sociodemographic characteristics of healthcare providers in health centers (HCs) in the South Gondar Zone, Amhara, Ethiopia, 2022**.**

Variable	Frequency	Percent
Sex		
Male	246	59.0
Female	171	41.0
Age		
25−30	227	54.4
31–35	125	30.0
36–40	55	13.2
≧41	10	2.4
Religion		
Orthodox	360	86.3
Muslim	50	12.0
Protestant	6	1.4
Others	1	0.2
Educational status		
BSc	165	39.6
Diploma	245	58.8
Others	7	1.7
Ethnicity		
Amhara	412	98.8
Oromo	4	1
Tigray	1	0.2

BSc, Bachelor of Science.

### Work experience and training in the IMNCI protocol

Regarding IMNCI training, only 79 (18.9%) of the respondents attended at different periods. Of the total, more than half 55 (69.6%) of the respondents had attended in-service training and 24 (30.4%) had received preservice training. Most employees received training for 7 days (25, 31.6%) and 10 days (17, 21.5%). Among the HCW who received training, 73(92.4%) did not undergo follow-up training (Table 3). From in-depth interview (IDI) evidence all participants indicate that a lack of IMNCI training among HCW contributes to poor usage of the IMNCI protocol.

**Table 3:** Work experience and types of training of healthcare workers (HCW) in the South Gondar Zone, Amhara, 2022 (*n* = 417).

Variable	Frequency	Percent
Service year		
<5 years	185	44.4
5–10 years	177	42.4
10–15	40	9.6
>15 years	15	3.6
Serving in OPD		
0–5 years	255	61.2
6–10 years	138	33.1
>10 years	24	5.8
Attained training		
Yes	79	18.9
No	338	81.1
Types of training		
Preservice	24	30.4
In-service	55	69.6

OPD, outpatient department.

A 29-year-old male clinical nurse stated the following:

I am the one who took IMNCI training but it does not mean always online in treating the patient in under five OPD. When I off work others treat traditionally rather than classify and treat the patient according to the IMNCI protocol…. (Participant 2)

The participants expressed the benefit and necessity of follow-up training for better utilization of the IMNCI protocol and refreshing their knowledge:

I was taken IMNCI training since 2019 so as to boost knowledge on IMCI there must be follow up training to update the former information about IMNCI. (Participant 2)

Additionally, 185 (44.4%) participants had work experience <5 years, ∼255 (61.2%) participants had worked in under-five OPD for <5 years, and 138 (33.1%) participants had worked for >5 years (Table 3).

## Practice of IMNCI case management protocol

Of the six steps of the IMNCI case management protocol, more than half of the respondents, 237 (56.8%), 240 (57.6%), and 101 (50.2%) provided counsel caretakers, provided follow-up care, and treated the child as a difficult step to practice. Among the six steps of the IMNCI protocol, assessing the child’s condition and classifying the illness were the most difficult steps to practice [130 (31.2%) and 118 (28.3%), respectively (Table 4)].

**Table 4:** Steps in the case management protocol by healthcare workers (HCW) in the South Gondar Zone, Amhara, 2022. (*n* = 417).

Variables	Category	Frequency	Percent
Assess the child's condition	Always difficult	130	31.2
	Sometimes difficult	136	32.6
	Not difficult	151	36.2
Classify the illness	Always difficult	118	28.3
	Sometimes difficult	118	28.3
	Not difficult	181	43.4
Identify treatment	Always difficult	84	20.1
	Sometimes difficult	129	30.9
	Not difficult	204	48.9
Treat the child	Always difficult	90	21.6
	Sometimes difficult	118	28.3
	Not difficult	209	50.1
Counsel caretaker	Always difficult’	79	18.9
	Sometimes difficult	101	24.2
	Not difficult	237	56.8
Provide follow-up care	Always	74	17.7
	Sometimes difficult	103	24.7
	Not difficult	240	57.6

### HCW performance of the IMNCI protocol

The majority of the study participants, 278 (66.7%), 260 (62.4%), 330 (79.1%), 277 (66.4%), 205 (49.2%), and 268 (64.3%), consistently checked for vaccination, pallor, diarrhea, weighing children, comparing weight against the chart, and examining for ear problems, respectively (Table 5). This finding was supported by the lack of IMNCI materials, particularly unavailability of weighing scales, which resulted in underutilization of the protocol.

**Table 5:** IMNCI activity performance of the study participants of the South Gondar Zone, Amhara, Ethiopia, 2022.

Variables	Category	Frequency	Percent
Checking for danger signs	Always	399	95.7
	Sometimes	18	4.3
	Not performed	0	0
Checking for vaccination	Always	278	66.7
	Sometimes	139	33.3
	Not performed	0	0
Assessing pallor	Always	260	62.4
	Sometimes	157	37.6
	Not performed	0	0
Assessing fever	Always	369	88.5
	Sometimes	48	11.5
	Not performed	0	0
Assessing diarrhea	Always	338	81.1
	Sometimes	79	18.9
	Not performed	0	0
Assessing malaria	Always	330	79.1
	Sometimes	87	20.9
	Not performed	0	0
Assessing cough	Always	372	89.2
	Sometimes	45	10.8
	Not performed	0	0
Weighing the children	Always	277	66.4
	Sometimes	140	33.6
	Not performed	0	0
Checking weight against the chart	Always	205	49.2
	Sometimes	212	50.8
	Not performed	0	0
Checking for ear problems	Always	268	64.3
	Sometimes	149	35.7
	Not performed	0	0

IMNCI, Integrated Management of Newborn and Childhood Illness.

A 31-year-old BSc nurse from the Farta district stated the following:

…in the presence of more patients, not only my partners but also me not took weight and not compare with the chart. It is the main challenge for us. (Participant 6)

## Time spent while using the IMNCI case management protocol

Regarding the time spent, 62 (14.9%) and 198(47.5) respondents strongly agreed and strongly disagreed, respectively, that they spent more than 1 h using the protocol. Most of the respondents (184, 44.1%) agreed to spend between 30 and 40 min using the IMNCI protocol. A total of 134 respondents strongly agreed on the time taken while using the IMNCI protocol for 15–20 min, and 146 respondents agreed on the time taken while using the IMNCI protocol for a single case.

Only 108 (25.9%) of the respondents strongly agreed that the time taken was up to 14 min, while 130 (31.2%) strongly disagreed with managing a case using the IMNCI protocol. Participants acknowledged that IMNCI took more than 20 min, which is above the WHO range:

It is difficult to work always as per the guideline. If you have 40 patients and you investigate them as per the protocol, you only treat 3 to 4 patents. IMNCI is time consuming. (Male, BSc nurse from the Dera district, Participant 5)

Most HCW did not use the protocol while treating fewer than five patients. However, the WHO recommends using the IMNCI protocol only to manage infant cases.

Approximately 60.4% of the respondents mentioned that the IMNCI protocol was time-consuming. Similarly, 56.6% of the HCW reported that the IMNCI protocol is tedious. Most of the participants mentioned in the IDI report that they find IMNCI to be time-consuming and tedious, which discourages its practice among the untrained ones. Interviews conducted with all trained HCW showed that they used the guidelines; however, their implementation was influenced by the high outpatient flow in their HC.

A 30-year-old male clinical nurse from the Fogera district stated the following:

“IMNCI is tedious to manage one case we took history from the mother about illness, vaccination status, vitamin A, deworming and took vital sign from the child that why IMNCI is tedious. Sometimes some patients may not be seen by a nurse due to patient overload. we share the card to other adult OPD to manage them without IMCNCI protocol…. (Participant 1)

### Leadership-related factors

Approximately 53.7% of the respondents mentioned that a lack of supervision affected their utilization of the IMNCI protocol while managing ill children. Most of the time, the Woreda Health Office did not provide supportive supervision regarding the IMNCI issue. As participants quoted, IMNCI is mostly forgotten by the management staff, which is why the level of utilization is low. A 27-year-old female clinical nurse from the Fogera district stated the following:

No one help you with related to IMNCI including the Woreda Health Office. No one comes and supervises you whether you work according to the protocol or not you yourself know whether you work according to the training and protocol. There is no regular supervision, even though they become fault finders rather than filling the gaps identified. Supervisors did not show the way forward only they said why you did not do so. (Participant 4)

The head of the HC and the Woreda Health Office pay less attention to IMNCI than to monthly reportable programs such as maternal and tuberculosis issues.

## Availability of resources to the IMNCI protocol

According to this study, respondents reported unavailability of booklets (50.4%), unavailability of medications (54.4%), and lack of equipment (52%) as resource limitations impacting the utilization of IMNCI in practice.

The absence of IMCNCI equipment and drugs is the main challenge for HCW in practicing the IMNCI protocol during their daily duties. The IMNCI focus in this study was the opinion that HCs left the logistic aspect for the proper utilization of the IMCI protocol. Consumables such as supplies, documentation, and reporting tools and drugs were mentioned by HCW as inadequate at the HC level:

“IMNCI drugs frequently stocked out which affect us to treat the ill according to the IMNCI protocol. The IMNCI drug was the most important determinant for utilizing the IMNCI protocol. Most of the time, the IMNCI chart booklet and register not avail in that case we use the adult patient register instead of the IMNCI protocol''. (29 years, male, health officer from Este district, participant 3)

Another 30-year-old clinical nurse from the Fogera district, an IMNCI focal person, stated the following:

Most of the time IMNCI drugs stock out. This results in neglect the IMNCI protocol and treat the patient with other unrecommended drugs like cotrimoxazole. However, the new guidelines avoid cotrimoxazole, which is replaced by amoxicillin. When amoxicillin was stocked out, cotrimoxazole was used. (Participant 1)

Approximately 56.8% of the respondents agreed that patient overload affects the implementation of all the contents of the IMNCI protocol. A negative attitude was also a barrier to the utilization of the IMNCI protocol. Qualitative evidence showed that most trained individuals had a good attitude toward the benefit of maintaining the IMNCI protocol.

A 28-year-old IMNCI focal participant from the Fogera district further stated the following:

“......Ended IIMNCI is good for knowledge. Some employees were depressed when they heard IMNCI. Some employees even frustrated working with IMNCIs”. (Participant 1)

Another 31-year-old IMNCI focal person from Fogera district stated the following:

“…… Employees in our health center did not have a positive attitude toward IMNCIs. Most employees did not like the IMNCI. Except for trained persons, none used a chart booklet in our health center”. (participant 4)

### Utilization status of IMNCI by health professionals

The study findings showed that the proportion of IMNCI utilization was 63.1% (CI 95%: 58.2%, 67.7%).

### Factors associated with the utilization of the IMNCI protocol

Bivariable analysis showed a statistically significant association between IMNCI protocol utilization and receiving IMNCI training, referring to a chart booklet during the case management process. A high patient–nurse ratio, lack of a good attitude toward IMNCI, unavailability of an IMNCI chart booklet, unavailability of IMNCI drugs, lack of supervision, and lack of IMNCI equipment were candidate variables for multivariable logistic regression analysis. Of the candidate variables for multivariate analysis, only four were found to be significantly associated with the utilization of IMNCI protocols. The unavailability of the IMNCI chart booklet, unavailability of IMNCI drugs, IMNCI training, and referring to the chart booklet during the case management process were significantly associated with IMNCI utilization. The odds of IMNCI utilization among those HCW who underwent IMNCI training was 3.13 times higher than that of those who did not take IMNCI training [adjusted odds ratio (AOR) = 3.13, 95% CI: 1.594, 6.147]. The unavailability of the IMNCI chart booklet reduced the utilization of the IMNCI protocol by 70.1% compared with the presence of the IMNCI chart booklet (AOR = 0.299, 95% CI: 0.156, 0.571). The lack of IMNCI drugs reduced the utilization of the IMNCI protocol by 75.7% compared with the presence of IMNCI drugs (AOR = 0.243, CI: 95%:0.128, 0.464). HCW who always referred to chart booklets during case management were 3.34 times more likely to utilize IMNCI protocols (AOR = 3.34, 95% CI: 1.99, 5.60) than those who did not refer to the chart (see Table 6).

**Table 6:** Bivariable and multivariable binary logistic regression analysis for factors associated with the utilization status of the IMNCI protocol among health professionals in the South Gondar Zone, Amhara, Ethiopia, 2022.

Variable	Category	Level of IMNCI utilization				
		High level	Low level	COR (95% CI)	AOR (95% CI)	*p*-value
Attended IMNCI training	Yes	63	16	2.72 (1.51, 4.90)	3.130 (1.594, 6.147)	**0.001**
	No	200	138	1	1	
Referring to the chart booklet during case management process	Yes	184	67	3.02 (2.00, 4.57)	3.34 (1.99, 5.60)	**0.000**
	No	79	87	1		
High patient–nurse ratio	Yes	116	121	0.215 (0.136, 0.339)	0.725 (0.384, 1.370)	0.322
	No	147	33	1		
Unavailability of the IMNCI chart booklet	Yes	85	125	0.111 (0.069, 0.179)	0.299 (0.156, 0.571)	**0.000**
	No	178	29	1		
Unavailability of IMNCI drug	Yes	98	129	0.115 (0.070, 0.189)	0.243 (0.128, 0.464)	**0.000**
	No	165	25	1	1	
Lack of supervision	Yes	123	101	0.461 (0.306, 0.696)	1.224 (0.690, 2.171)	0.488
	No	140	53	1		
Lack of IMNCI equipment	Yes	102	115	0.215 (0.138, 0.334)	0.680 (0.364, 1.270)	0.227
	No	161	39	1		

IMNCI, Integrated Management of Newborn and Childhood Illness; AOR, adjusted odds ratio. A *p*-value < 0.05 was considered as significant.

Statistically significant values are in bold.

## Discussion

This study aimed to identify the utilization of IMNCI protocol and associated factors among HCW in the South Gondar Zone, Amhara, Ethiopia. The study findings showed that the proportion of IMNCI utilization was 63.1% (95% CI: 58.2%, 67.7%). This is below the WHO standard level, which is 68% or above (5). This rate is higher than that reported in a study conducted in the Oromia region, which showed that the overall IMNCI guideline utilization was 58.7% (6). This is also higher than that in a study conducted in Kenya, in which utilization of the IMNCI protocol by health professionals was only 42% (7), and in Nigeria, in which the overall IMNCI utilization was 53.8% (8). This difference might be due to the increased quality of service provided by health institutions, IMNCI equipment availability, IMNCI training status, and the time of the study conducted. This is higher than a study in Benin, in which the proportion of utilization of the guideline was 15% (9). This discrepancy might be due to the time of the study being conducted in the country. This study showed that factors such as lack of chart booklets (50.4%), lack of IMNCI equipment (52%), lack of supervision (53.7%), unavailability of IMNCI drugs (54.4%), high patient–nurse ratio (56.8%), and staff turnover (52.8%) affected utilization of the IMNCI protocol. This is similar to the study in Butaleja which showed that supportive supervision, lack of IMNCI guidelines, lack of prereferral treatment for urgent referral, shortage of essential drugs lack of sound referral system affect the IMNCI strategy (10). This is higher than the study in West Arsi Zone, which lacked the necessary medications and supplies (37.3%) (6). This might be due to the difference in the ability to provide in the healthcare financing system, as per the standard. Both limited time and unavailability of the booklet could also impact the health professional's ability to track down the booklet.

HCW who always referred to chart booklets during case management were 3.34 times more likely to utilize IMNCI protocols than those who did not refer to charts. This might be because the chart boosts their knowledge and skills regarding how to utilize the protocol to manage the five cases faced by them. The odds of IMNCI utilization among HCW who received IMNCI training were 3.13 times higher than that among HCW who did not receive IMNCI training. This is similar to a study conducted in the Oromia region where providers who had attended training were 2.6 times more likely to utilize IMNCI guidelines than those who had not attended IMNCI training (5). This finding is also in line with those of a study conducted in Malawi (11, 12). Training is the most significant factor for IMNCI utilization; however, only 18.9% of HCW in the HC of four districts of the South Gondar Zone had received IMNCI training, which was less than the WHO recommendation that at least 60% of healthcare professionals who diagnose and treat children under five in the health facilities should receive training in the IMNCI guidelines. This study showed that only 7.6% of health professionals received follow-up training; however, follow-up training was designed to increase methods for skill reinforcement, record review, assessment, and enhancing job designs to encourage HCW, which is less than a study conducted in the Oromia region (6). This might be due to the poor attention given by the management staff for IMNCI follow-up training.

This study revealed that 49.2% of the employees measured and compared the weight of children using charts. This is somewhat higher than a study conducted in Uganda but the WHO recommends measuring the weight of all children to struggle with malnutrition since it is one of the reasons for the infant mortality increment (13).

This study revealed that some employees had a poor attitude toward the IMNCI protocol. This is inconsistent with studies in Tanzania, where most employees had good attitudes. This difference may be due to limitations in capacity-building and behavioral change initiatives conducted in the zone (5).

Sometimes, HCW are not able to prescribe drugs for the treatment of the disease as per the IMNCI protocol due to poor knowledge, attitudes, and resources. This is similar to a study conducted in Busolwe Hospital, Butaleja, and Ghana, in which cases were managed as specific disease conditions and rarely according to the IMNCI protocol (10, 14, 15). This study showed that managing under-five patients using IMCI is time-consuming and only a small number of cases will be seen. This is similar to a study in Botswana, where nurses who treat according to the protocol spend more time but spend more time than the WHO which is 15–20 min. This might be due to a lack of training in which training facilitates the time intake of case treatment by the IMNCI protocol (16). This study revealed that leadership and governance, mentoring, and supervision affect the performance of HCWs in the utilization of IMNCI. This is in line with a study conducted in South Africa (17).

## Limitations of the study

The results of this study rely on self-report from healthcare professionals, and as a result, there might be an influence of social desirability. This study was also limited to the South Gondar Zone cluster HCs, and this finding cannot be generalized to the Amhara region or the country level.

## Conclusion

This study revealed that utilization of the IMNCI protocol was low which is 63.1%. From this study, health system factor supervision, shortage of medical consumables and equipment, HCW factor attitude, and training were identified as factors that affect the utilization of the IMNCI protocol.

## Recommendation

**To the researchers:** This research could become better if further study is conducted at the regional level (large-scale study).

**To the HC:** The HC should strengthen the health systems and infrastructure to improve the accessibility and availability of essential resources (availability of booklets and drugs) needed to implement the IMNCI protocol.

**To the WHO:** The WHO should also provide training to HCW to increase awareness among healthcare providers and communities about the benefit of using the IMNCI protocol for early detection and management of childhood illnesses. The office should also increase regular supportive supervision and monitoring and evaluate the implementation of the IMNCI protocol to identify gaps and challenges and make necessary adjustments to improve its utilization.

## Data Availability

The original contributions presented in the study are included in the article/Supplementary Material, further inquiries can be directed to the corresponding author.
